# Millimeter-Scale Magnetic Positioning Using a Single AMR Sensor and BP Neural Network

**DOI:** 10.3390/s26041339

**Published:** 2026-02-19

**Authors:** Guanjun Zhang, Zihe Zhao, Peiwen Luo, Wanli Zhang, Wenxu Zhang

**Affiliations:** National Key Laboratory of Electronic Thin Films and Integrated Devices, University of Electronic Science and Technology of China, Chengdu 611731, China; 202322310221@std.uestc.edu.cn (G.Z.); zihezhao@std.uestc.edu.cn (Z.Z.); 202311311103@std.uestc.edu.cn (P.L.); wlzhang@usetc.edu.cn (W.Z.)

**Keywords:** magnetic field positioning, back propagation neural network, magnetic fields sensor

## Abstract

Unlike conventional positioning systems that rely on multiple sensors, the positioning system proposed in this study uses a single anisotropic magnetoresistive (AMR) sensor to measure the magnetic field of a target permanent magnet. This approach significantly reduces the system hardware cost and complexity, facilitating the miniaturization of positioning systems. Leveraging a BP neural network model, which is shown to be fast and accurate, the positioning system obtains the real-time magnetic field of the target magnet using a single sensor, subsequently converting three-axis magnetic field data into coordinate information to achieve real-time tracking and localization. The results show that the root mean square errors (RMSEs) for the X and Z axes in the simulation are 0.27 mm and 0.26 mm, respectively, while the RMSEs for the X, Y, and Z axes in the actual test are 0.83 mm, 1.15 mm, and 0.85 mm, respectively. It is also observed that the positioning error correlates with variations in the magnetic field with respect to position, which originate from the strong distance-dependent nonlinearity of the magnetic field. This method not only reduces hardware costs but also maintains accuracy. It is particularly well-suited to applications requiring high-precision positioning and tracking, achieving millimeter-level accuracy within a volume of 50 × 40 × 40 mm^3^. It has potential applications in aerospace intelligent connectors, medical devices and automation systems, where space and signal lines are limited.

## 1. Introduction

With the rapid advancement of industrial automation, medical imaging, aerospace, and other fields, precise location information acquisition has become increasingly important. The acquisition of position information relies heavily on real-time position and distance measurements. Magnetic field-based sensor positioning technology is characterized by its lightweight, compact size and low power consumption. Additionally, because magnetic fields are unaffected by external environmental factors, magnetic field positioning technology has become invaluable across many fields. Furthermore, using a permanent magnet as a passive magnetic source enhances the energy efficiency and cost-effectiveness of the positioning system. Positioning technology based on magnetic fields is primarily utilized in indoor positioning [1,2,3], medical applications [4,5,6,7], environmental monitoring [8], military fields [9], and other research areas. In many application scenarios, such as spacecraft systems [10], devices are often required to be as small and light in weight as possible. A single-sensor configuration can reduce the number of sensors compared to sensor arrays, thereby minimizing space occupation and facilitating integration into compact devices. At the same time, it lowers hardware costs and reduces the workload of subsequent maintenance.

Magnetic field localization often leverages the sensitivity of modern solid-state sensors to detect minor variations in the measured vector flux density (MFD) caused by changes in the relative position between the magnetic source and the sensor. Common magnetic sensors used for positioning include Hall-effect, AMR, GMR, and TMR sensors. Among them, AMR sensors provide a good compromise between sensitivity, bandwidth, cost, and power consumption, making them suitable for compact positioning systems. A function that relates position to vector flux density is necessary to ascertain the position. However, the mapping of the magnetic fields and the positions is not trivial, and it is one of the critical problems to be solved in order to get a highly accurate position. Some widely used modeling methods [11] include single magnetic dipole (MD) and distributed multipole models. The magnetic dipole model simplifies the magnetic properties of materials to a pair of magnetic dipoles composed of an equal number of magnetic monopoles oriented in opposite directions [12]. These models characterize the magnetization of the system using magnetic moments. The distributed multipole model describes the distribution of electric or magnetic fields in complex physical systems. It is an extension [13] of the single magnetic dipole model, incorporating multiple poles to more accurately represent the field generated by the distribution of spatial charge or magnetic moments. However, a single magnetic dipole can only [14] describe the characteristics of the magnetic field at considerable distances from the source, and the model loses its accuracy as one approaches the magnet. The distributed multipole model consists of a spatial array of dipoles specifically designed to address the limitations of the magnetic dipole model; however, constructing an appropriate spatial dipole array can be quite cumbersome.

In addition to the analytical expressions above, the application of machine learning, neural networks, and other techniques in magnetic field positioning technology [15,16] is promising. Artificial neural networks (ANNs) offer distinct advantages in the real-time modeling of large datasets. Comparative analysis of positioning results from the nonlinear positioning algorithm and the BP neural network [17,18,19] indicates that the positioning accuracy of the BP neural network is superior. The artificial neural network (ANN) is influenced by biological neural networks, allowing it to combine the accuracy of the ANN model with the speed and range advantages of the single dipole model [20]. Sun et al. [21] proposed using convolutional neural networks (CNN) to classify floors and locations based on Bluetooth received signal strength (RSS). The magnetic field data are employed to calculate the final coordinates. This approach enables fingerprint positioning in large-area and multi-floor indoor environments, achieving an accuracy improvement of nearly 50% over traditional Bluetooth and magnetic fingerprint localization methods.

Compared to more complex models such as deep neural networks (DNN) and convolutional neural networks (CNN), the BP neural network has a lower computational complexity and can be effectively trained with fewer sample data. Therefore, this study combines a BP neural network with numerical magnetic field distributions to achieve positioning within a finite range.

In this work, a permanent magnet used a single magneto resistance (AMR) sensor to detect the local magnetic field, facilitating a low-cost and low-power positioning system. To provide training data for the neural network model, the finite element method (FEM) was used to calculate the magnetic field and convert it into corresponding coordinate data through a BP neural network. Section 2 introduces the overall scheme of the magnetic field positioning system, detailing the specifications of the permanent magnet, sensor selection, and the principles underlying the positioning method. Section 3 presents the permanent magnet positioning approach, including simulation modeling and the neural network training process. Section 4 evaluates the model derived from the neural network, encompassing both simulation and experimental tests, and an error analysis.

## 2. Overall Scheme

**Figure 1:**
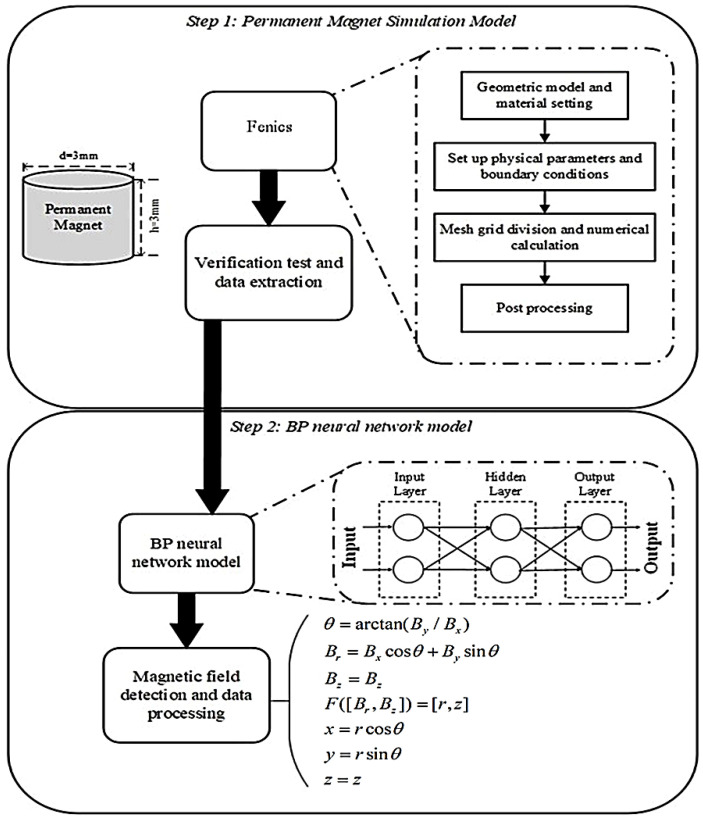
Flow chart of the overall scheme.

Using a permanent magnet in the positioning system can achieve significant energy savings and cost effectiveness. Typical shapes of permanent magnets include cylinders, spheres, and cubes. In this study, we selected an axially magnetized cylindrical permanent magnet with a diameter of 3 mm and a thickness of 3 mm. Finite element analysis software was used to establish the magnetic field model of the permanent magnet and perform the necessary calculations to obtain the spatial magnetic field distribution of the permanent magnet quickly and accurately. The overall scheme of this study is illustrated in Figure 1.

**Figure 2:**
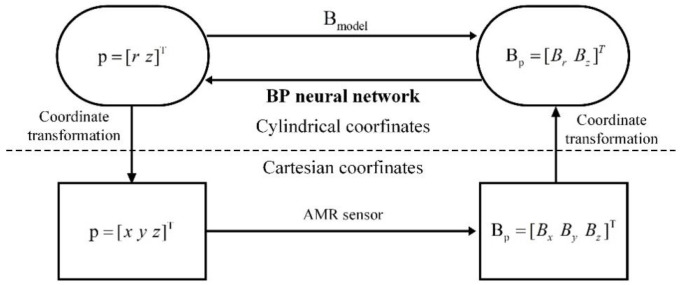
Mapping between spatial coordinates and the magnetic field.

The processes of mapping the magnetic field and coordinates are shown in Figure 2. Firstly, the external magnetic field distribution of the permanent magnet was calculated using a finite element method (Fenics), resulting in the magnetic field distributions Br and Bz at the corresponding positions within the cylindrical coordinate system. Subsequently, a BP neural network was employed to learn from the simulated magnetic field components Br and Bz, along with the corresponding positions in the cylindrical coordinate system. The trained neural network model was applied to the real-time detection system for the three-axis magnetic field of the permanent magnet. The magnetic field data obtained from the sensor were converted to the cylindrical coordinate system, where the neural network model was utilized to predict the r and z values. Ultimately, the coordinates x, y, and z were derived based on rotational symmetry.

An AMR sensor (MMC5603NJ, MEMSIC Semiconductor Co., Ltd., Tianjin, China) measured the magnetic field in three-dimensional space. The sensor has a full-scale measurement range of 30 Gauss, a resolution of up to 0.0625 mG/LSB in 20 bit operation mode, and a total RMS noise level of 2 mG. In this study, a sensor module consisting of one MMC5603NJ sensor was used to detect the magnetic field. According to the specified parameters, the distance from the surface of the magnet ranges from 12 mm to 65 mm in the axial direction and within ±20 mm in the radial direction.

**Figure 3:**
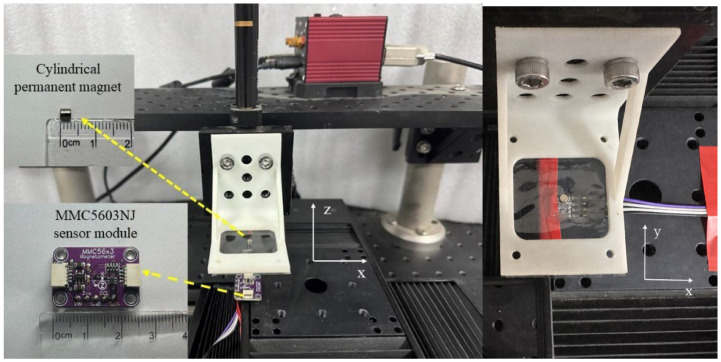
Physical diagram showing the system’s construction, the sensor module is located below the permanent magnet.

The positioning system including the permanent magnet and the sensor fixed on the motorized stage is shown in Figure 3. A computer can control the motorized stage, which moves along the x, y, and z axes in combination or in a single direction. The permanent magnet is fixed on a bracket connected to the motorized stage, and the sensor module is placed horizontally under the permanent magnet. The triaxial magnetic field strength measured by the sensor varies with the magnet’s position. These data are subsequently transmitted to a computer via a System-on-Chip Microcontroller for 32-bit ARM Cortex-M Architecture (STM32F103C8T6, STMicroelectronics N.V., Plan-les-Ouates, Switzerland), enabling the real-time display of position information and motion trajectory on the computer.

## 3. Positioning Methods

### 3.1. Magnetic Field Model

A cylindrical permanent magnet (NdFeB) with a diameter and thickness of 3 mm was used as the magnetic field source. The magnetization direction was along the *Z* axis. Due to the rotational symmetry of the system, a 2D model was used for simulation in this study. In the two-dimensional model, the magnetic field and position coordinates are expressed in a cylindrical coordinate system, and the relationship between them and the Cartesian coordinate system is as follows.

(1) $$\left\{\begin{array}{l} B_{r}=B_{x}\cos\theta +B_{y}\sin\theta \\ \theta =\arctan(B_{y}/B_{x})\\ B_{z}=B_{z} \end{array}\right.$$

(2) $$\left\{\begin{array}{c} x=r\cos\theta \\ y=r\sin\theta \\ z=z\;\;\; \end{array}\right.$$

Within a predefined and limited working volume, the magnetic field generated by the permanent magnet is a deterministic function of spatial position. Therefore, the three-axis magnetic field vector uniquely corresponds to a spatial location in this region. Moreover, by exploiting the cylindrical symmetry of the axially magnetized magnet and expressing the magnetic field in cylindrical coordinates, the solution space is further constrained, which effectively avoids ambiguity in the mapping between magnetic field and position.

**Figure 4:**
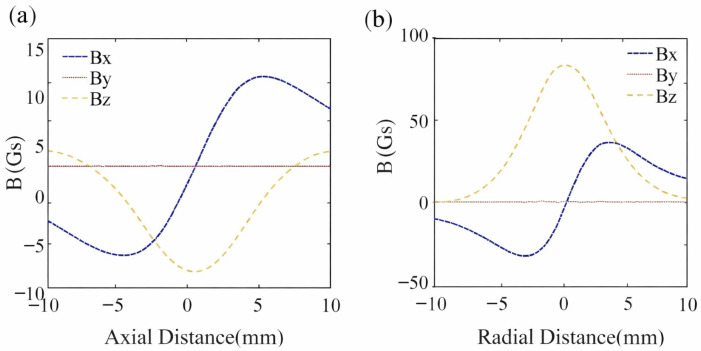
Variation in spatial magnetic field intensity of the cylindrical permanent magnet. (**a**) Axial path. (**b**) Radial path.

Taking the XOZ plane as an example, the spatial magnetic field intensity of the cylindrical permanent magnet varies along the axial and radial paths, as illustrated in Figure 4. It can be observed that the magnetic field components in the *X*-axis and *Y*-axis directions are distributed symmetrically about the center. In contrast, the magnetic field in the *Z*-axis direction exhibits axial symmetry.

When the distance is more than 2.5 times the target size, the target can be approximated as a magnetic dipole. Equation (3) shows the expression of the magnetic induction intensity of the magnetic field generated by the magnetic dipole at any point in space.

(3) $$\vec{B}=\frac{\mu_{0}}{4\pi }\left[\frac{3(\vec{M}\cdot \vec{n})\vec{n}-\vec{M}}{r^{3}}\right]$$

In (3), $\vec{n}$ represents the unit vector of the magnetic dipole, $\vec{M}$ is the magnetic moment of the magnetic dipole, and $\mu_{0}$ is the permeability of free space. To show that the dipole model cannot be used to model the magnetic field in the vicinity of the magnet, we measured $B_{z}$. B_z_ at z = 12.3 mm with x ranging from −10 mm to 10 mm and compared the actual measured value with the simulation result to verify the accuracy of the simulation.

**Figure 5:**
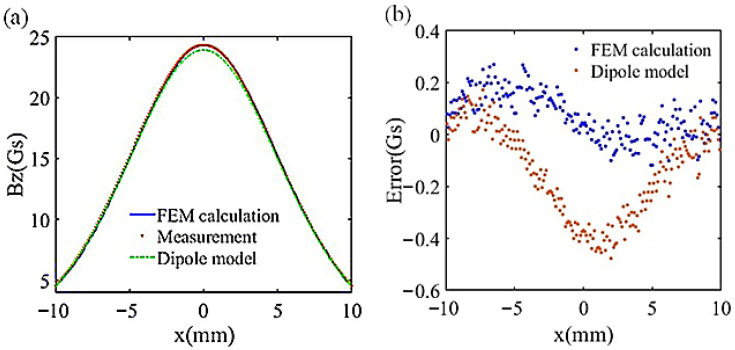
(**a**) Comparison of FEM calculation, measurement, and dipole model field distribution on the same path. (**b**) Error distribution of FEM calculation and dipole model. The error of the FEM calculation is smaller than that of the dipole model.

In the FEM calculation, the magnetization direction of the permanent magnet is oriented along the *Z* axis, and the distribution of the external magnetic field of the permanent magnet in the simulation can be adjusted by tuning the size of $M_{s}$. In Figure 5a, the actual magnetic field distribution is compared with the simulation model and the results of the magnetic dipole. The magnetic dipole model introduces positioning errors, requiring the distance from the test location to the magnet to significantly exceed the magnet’s dimensions. Therefore, the magnetic dipole model does not apply to the nearby field surrounding the magnet.

### 3.2. BP Neural Network and the Training Models

To accurately model the field distribution, we trained a numeric model from the data obtained by the FEM calculation instead of using a dipole model. The back propagation (BP) neural network was used to establish the mapping between magnetic field strength and the corresponding positions. As a robust nonlinear prediction method, the BP neural network demonstrates excellent nonlinear fitting and generalization capabilities, enabling it to efficiently and accurately predict complex data. The basic structure of the BP neural network consists of an input layer, a hidden layer and an output layer, as shown in Figure 1. The forward propagation formula is as follows.

(4) $$z_{j}^{l}=\sum_{i=1}^{n_{l-1}}w_{ji}^{l}a_{i}^{l-1}+b_{j}^{l}$$

(5) $$a_{j}^{l}=f(z_{j}^{l})$$

where $z_{j}^{l}$ is the weighted input of the L-layer and the J-th neuron; $w_{ji}^{l}$ is the weight between the L-th and J-th neurons and the L-th and I-th neurons; $b_{j}^{l}$ is the bias of the j-th neuron in the L layer; $a_{i}^{l-1}$ is the activation value of the I-th neuron in L1 layer. The f(x) is the activation function, and the activation function of hidden layer neurons can be defined as the hyperbolic tangent function, and the expression is

(6) $$f_{1}(x)=\frac{2}{1+\exp(-2x)}-1$$

The activation function of the neurons in the output layer can be expressed as a linear function:

(7) $$f_{2}(x)=x$$

The transverse magnetic field component (Br) and the longitudinal magnetic field component (Bz) at each point along the X, Y, and Z axes, measured at 0.1 mm intervals, are designated as the inputs to the neural network. At the same time, their corresponding positions are set as the outputs. Input data are normalized to a range of [−1, 1] to mitigate the impact of data size changes on network learning.

(8) $$X^{\prime }=\frac{X-\bar{X}}{X_{\max}-X_{\min}}$$

In this study, min–max normalization was performed using the global minimum and maximum values of each magnetic field component obtained from the training dataset. Specifically, the parameters $X_{\min}$ and $X_{\max}$ in Equation (8) were calculated from all FEM-generated samples and remained fixed during inference.

Before practical measurements, a calibration procedure was performed to align the sensor output with the training data range. During the calibration process, the environmental magnetic field was measured, and the sensor offset and proportional coefficient were adjusted to ensure that the normalized inputs fell within the interval [−1, 1].

In neural network training, the expression of mean square error (MSE) was used to measure the mean square deviation between the estimated value and the actual value of the model. The formula for MSE is as follows.

(9) $$\mathrm{MSE}=\frac{1}{N}\sum_{i=1}^{N}(a_{i}-e_{i}{)}^{2}$$

where n is the total number of samples, $a_{i}$ is the actual value of the i-th sample, and $e_{i}$ is the estimated value of the i-th sample.

**Figure 6:**
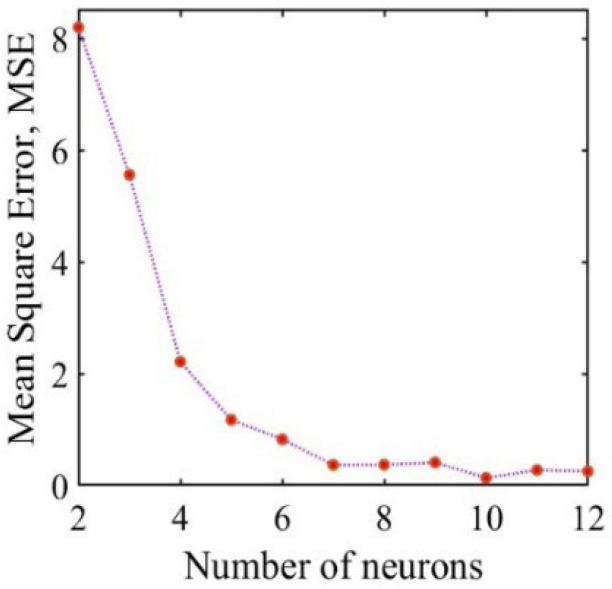
Convergence results of mean-square error for the BP neural network model.

In a BP neural network, the number of nodes in the hidden layer significantly influences the training outcomes of the model. The optimal number of hidden layer nodes can be determined based on the empirical formula.

(10) $$H=\sqrt{I+O}+k$$

where H represents the number of nodes in the hidden layer, I denotes the number of nodes in the input layer (i.e., the number of features), O indicates the number of nodes in the output layer, and k is a constant scaling factor, which is an integer in the range of 0–10. The number of nodes in the hidden layer can, therefore, be determined to be within the range of 2–12. Various configurations of hidden layer nodes were applied during the neural network training, as illustrated in Figure 6. The mean square error (MSE) was minimized when the number of hidden layer nodes was set to 10; thus, H is equal to 10.

**Figure 7:**
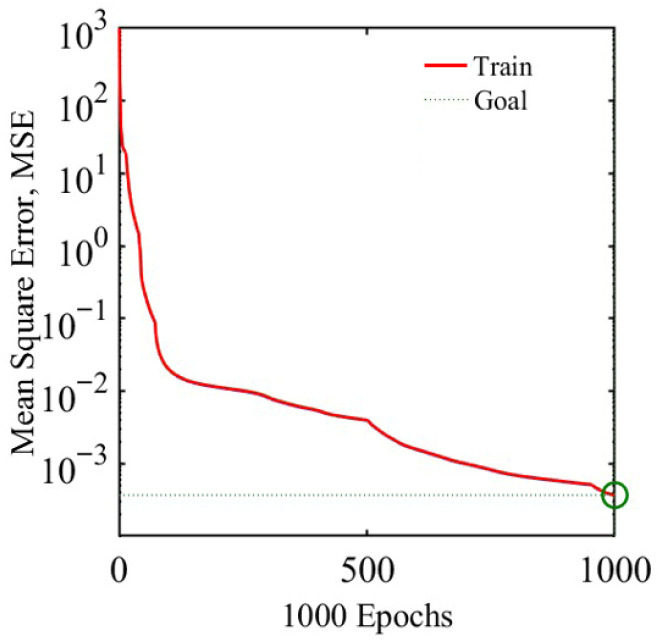
The training state diagram intuitively shows the changing trend in the training process.

**Figure 8:**
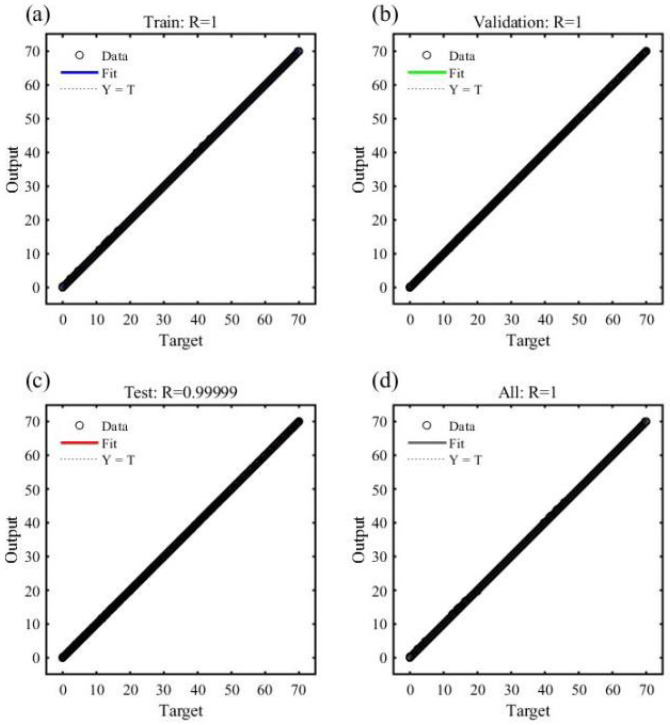
The regression plot shows the correlation between the prediction and the target output. (**a**) Training set; (**b**) Validation set; (**c**) Test set; (**d**) All the data.

**Figure 9:**
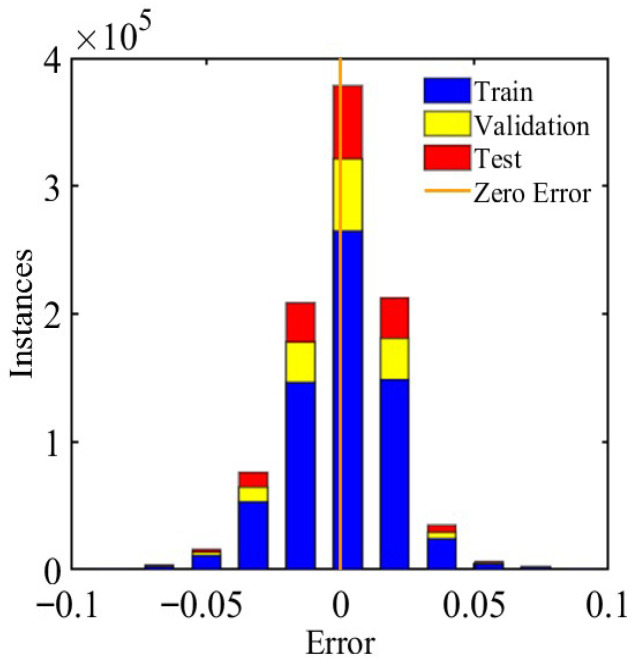
The error histogram reflects the frequency distribution of network prediction errors.

In the process of building the neural network, a second hidden layer containing five nodes was introduced to enhance the ability of the neural network to capture nonlinear relations to obtain high positioning accuracy. The sample data were divided into a training set, a validation set, and a test set of 70%, 15%, and 15%, respectively. The model was trained for 1000 iterations. The ANN model’s training performance plots, regression plots, and error histograms are presented in Figure 7, Figure 8 and Figure 9. The results indicate optimal performance after 1000 training iterations, yielding an MSE of 0.0036725. Most samples exhibit errors within the −0.5–0.5mm range. The correlation coefficient R for the training, validation and test sets is 1, demonstrating a strong correlation between the predicted values and the output data.

## 4. Analysis of the Positioning System

### 4.1. Position with Calculated Magnetic Field

**Figure 10:**
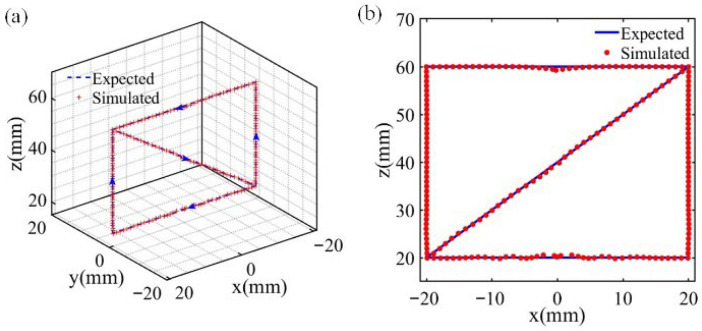
Simulation test results. (**a**) 3D view. (**b**) XOZ planar view. The blue arrow indicates the direction of movement.

Given that the cylindrical permanent magnet exhibits symmetry along the magnetization direction, the testing path was established in the XOZ plane, with the path range set within 40 × 40 mm^2^. The positioning path is defined by the coordinates (−20, 0, 0), (−20, 0, 60), (20, 0, 60), (−20, 0, 20), (20, 0, 20), and (20, 0, 60) in sequence with a step of 1 mm. In order to align the magnet to the global *Z* axis, we set up a criterion for *Bϕ* = −*Bx* sin(*θ*) + *By* cos(*θ*), which was less than 0.1. Figure 10 illustrates the positioning results in 3D space and in the XOZ planar view, showing that the moving path is as expected.

This study employed the root mean square error (RMSE) to represent the positioning error, which is calculated as

(11) $$\mathrm{RMSE}=\sqrt{\frac{1}{n}\sum_{i=1}^{n}\left[{\left(\Delta x\right)}^{2}+{\left(\Delta y\right)}^{2}+{\left(\Delta z\right)}^{2}\right]}$$

(12) $$\left\{\begin{array}{c} \Delta x=x_{i}-{x_{i}}^{\prime }\\ \Delta y=y_{i}-{y_{i}}^{\prime }\\ \Delta z=z_{i}-{z_{i}}^{\prime } \end{array}\right.$$

**Figure 11:**
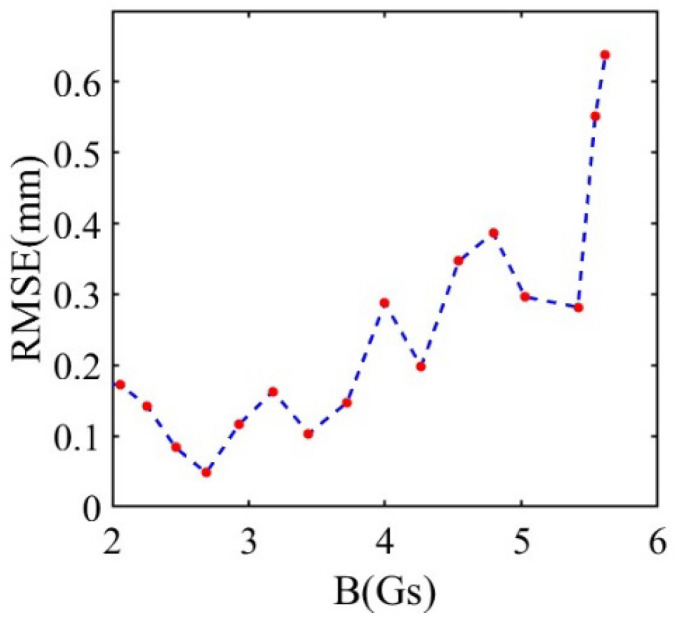
The curve of the RMSE as total magnetic field strength B increased in the simulation test.

The Δx, Δy, and Δz represent the errors between the actual ($x_{i}$, $y_{i}$, and $z_{i}$) and predicted values (${x_{i}}^{\prime }$, ${y_{i}}^{\prime }$ and ${z_{i}}^{\prime }$) along the X, Y, and Z axes, respectively, and n is the total number of data points in (7) and (8). Figure 11 shows the change in RMSE with the increase in magnetic field B in the simulation test. It can be seen that RMSE increases with the increase in the total magnetic field strength B. This is due to the fact that the field strength is dependent on the distance from the source as $r^{-3}$. When the field is large, it is more sensitive to the uncertainties of the positions. Based on the experimental data, the RMSE for the radial coordinates (X and Y coordinates) and the axial coordinates (Z coordinates) are 0.27 mm and 0.26 mm, respectively.

In the simulated environment, the sources of error primarily arose from the simplifications and assumptions inherent in the model, which reflect the system’s performance under ideal conditions without any dependence on the sensors and hardware.

### 4.2. Position with Measured Magnetic Field

**Figure 12:**
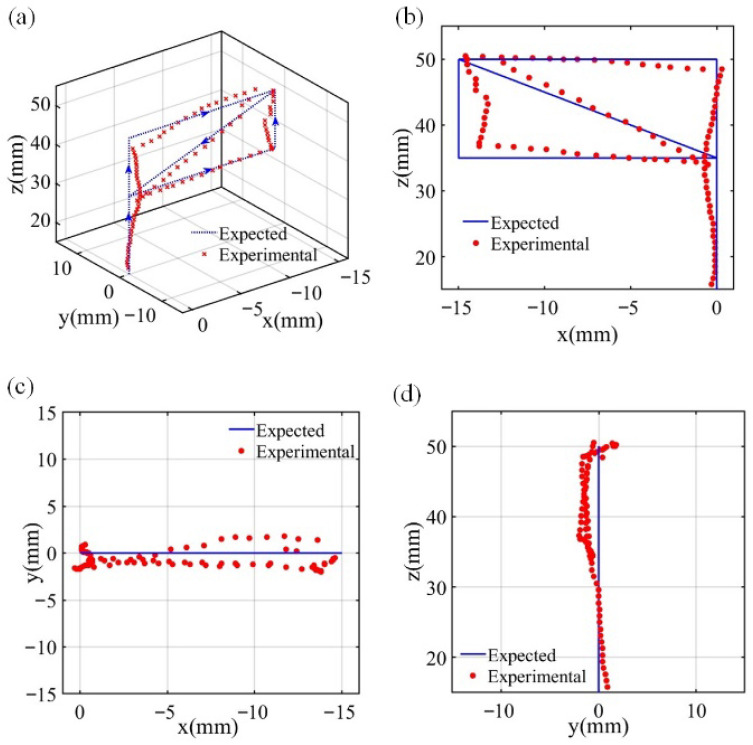
Experimental results. (**a**) 3D view (**b**) XOZ planar view (**c**) XOY planar view (**d**) YOZ planar view. The blue arrow indicates the direction of movement.

In the permanent magnet positioning system, in addition to errors arising from mapping the magnetic field to the positions, another significant source of error is the measurements of the magnetic field. Before practical measurements, the environmental magnetic field was measured and used for calibration to compensate for background offsets. Moreover, a magnetic shielding enclosure was applied during the experiments to suppress external magnetic interference, so that the influence of ambient magnetic noise on the positioning results was limited. The experiment also established a path in the XOZ plane, ranging between 20 mm and 40 mm. The positioning path is defined by the coordinates (0, 0, 15), (0, 0, 50), (−15, 0, 50), (0, 0, 35), (−15, 0, 35), and (−15, 0, 50) in sequence with a step of 1 mm. The three-dimensional representation of the experimental results is shown in Figure 12, where the expected path deviates slightly from the actual experimental results. As the X coordinate moves away from the origin, the error in the Z coordinate first decreases and then increases. Conversely, with an increase in the Z coordinate, the error in the Z coordinate initially increases before subsequently decreasing. The RMSEs of the X, Y, and Z axes are 0.83 mm, 1.15 mm, and 0.85 mm, respectively.

**Figure 13:**
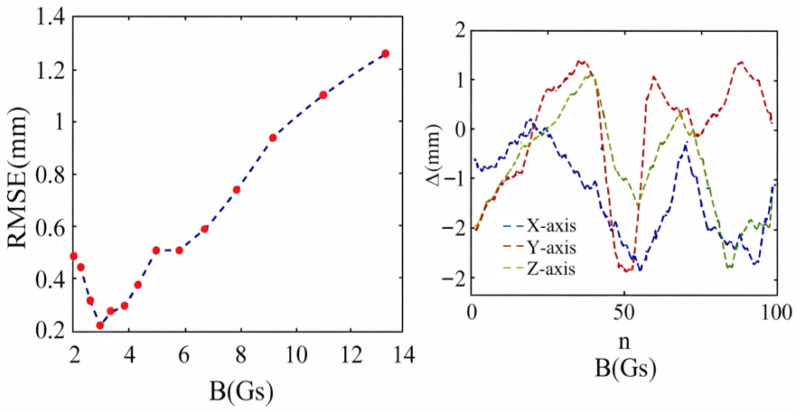
The (**left**) figure demonstrates that the curve of RMSE changes with the increase in total magnetic field strength in the actual test. The (**right**) figure represents the error curve of the X, Y and Z axes.

We can see that the maximum number of errors and RMSE increased almost five times from the simulations to the measurements. We observed that the errors around the magnet were low in the initial 30 steps (within 30 mm). This means that the errors come mainly from the sensor at this stage. Figure 13 illustrates that the error curves of the X, Y, and Z axes are irregular, and the RMSE showed an overall trend with increased total magnetic field strength B in the actual test. This is consistent with the simulation test results shown in Figure 11, which shows that the system error comes from the neural network model when B increases. This increment of the error with the intensity of the field is due to the nonlinearity of the function of the magnetic field and the distance. This is demonstrated by the simulated positioning shown in Figure 11 and was captured in the experiments. In order to reduce this error, the design of the field distribution should be optimized. A possible solution is to use multiple magnets to produce a magnetic field which is less sensitive to distances.

Magnetic-field positioning technology has been extensively studied for its potential in various applications. Magnetic-field-based positioning combined with machine learning has been widely investigated for indoor localization, medical tracking, and robotic applications [15,16,17,18,19,20,21,22], demonstrating that data-driven approaches can effectively handle nonlinear mapping between magnetic fields and positions. The positioning method proposed in this paper uses only one magnetic field sensor, ensuring positioning accuracy and at a low cost with low power consumption. A comparison with other magnetic field positioning studies is shown in Table 1.

The position estimation error in a cubic space of 2 × 2 × 2 m^3^ can be maintained within 10 cm using four sensors in the machine learning method [22]. In [23], a static tracking system composed of eight Hall sensors was used to locate objects in the body, which achieved an average position error of 2.04 mm within the 600 × 500 mm^2^ range. Similarly, the approach in [24] employed 64 sensors to achieve an error of 2.1 mm within a 500 × 500 × 500 mm^3^ range, with a positioning error of 2.1 mm. The positioning system described in this study only uses a single sensor, achieving an error of less than 1.2 mm within a 50 × 40 × 40 mm^3^ range. It can be seen that this study maintains positioning accuracy while reducing the number of sensors, lowering costs and power consumption. However, the positioning range is also reduced due to the sensitivity limit of a single magnetic field sensor. In this case, this one-sensor–one-magnet scheme is suitable for positioning in applications such as robot joints where space is limited or connectors with limited signal lines.

## 5. Conclusions

This paper has presented a magnetic-field-sensor-based approach for real-time positioning, utilizing a combination of magnetic field distributions and a BP neural network. The BP neural network model comprises two hidden layers, with the magnetic field distribution and corresponding position information of the simulated permanent magnet serving as the model’s input and output, respectively. The positioning performance was analyzed, demonstrating that the maximum RMSE of the positioning system was 0.27 mm in simulated tests and 1.15 mm in experiments. The results further indicate that the positioning error was mainly caused by the nonlinear relationship between the magnetic field and the distance. By employing FEM-generated magnetic field data for training, together with sensor calibration and magnetic shielding during experiments, the proposed system achieved stable and reliable positioning within a limited working range. This method satisfies the requirements for real-time operation and high precision and achieves millimeter-level accuracy while using only a single magnetic sensor. The positioning system offers advantages such as compact size, low cost, and simple hardware configuration, and can continuously monitor the target permanent magnet’s triaxial magnetic field, enabling real-time position updates and accurate tracking.

## Figures and Tables

**Table 1:** Comparisons of Magnetic Field Positioning Schemes.

	No. Sensors	Sensor Type	Method	Tracking Range (mm)	Maximum Position Error (mm)	Relative Error
This work	1	AMR sensor	BP Neural Network	50	1.15	2.3%
[22]	4	Three-axis magnetic-field sensor	Supervised Learning	2000	100	5.0%
[23]	8	Hall sensor	Levenberg–Marquardt (LM)	600	2.04	0.41%
[24]	64	-	PSO + LM	500	2.1	0.42%

## Data Availability

The raw data supporting the conclusions of this article will be made available by the authors on request.

## References

[1] Pasku V., De Angelis A., De Angelis G., Arumugam D.D., Dionigi M., Carbone P., Moschitta A., Ricketts D.S. (2017). Magnetic Field-Based Positioning Systems. IEEE Commun. Surv. Tutor..

[2] Sun M., Wang Y., Joseph W., Plets D. (2022). Indoor Localization Using Mind Evolutionary Algorithm-Based Geomagnetic Positioning and Smartphone IMU Sensors. IEEE Sens. J..

[3] Yeh S., Chiu H., Kao C., Wang C. (2023). A Performance Improvement for Indoor Positioning Systems Using Earth’s Magnetic Field. Sensors.

[4] Kim S., Bae S., Lee W., Jang G. (2024). Magnetic Navigation System Composed of Dual Permanent Magnets for Accurate Position and Posture Control of a Capsule Endoscope. IEEE Trans. Ind. Electron..

[5] Wang M., Song S., Liu J., Meng M.Q.H. (2021). Multipoint Simultaneous Tracking of Wireless Capsule Endoscope Using Magnetic Sensor Array. IEEE Trans. Instrum. Meas..

[6] Fu Q., Fan C., Wang X., Zhang S., Zhang X., Guo J., Guo S. (2021). A Compensation Method for Magnetic Localization on Capsule Robot in Medical Application. IEEE Sens. J..

[7] Liu S., Kim J., Kang B., Choi E., Hong A., Park J.-O., Kim C.-S. (2020). Three-Dimensional Localization of a Robotic Capsule Endoscope Using Magnetoquasistatic Field. IEEE Access.

[8] Wu Z., Huang H., Zhao G., Liu J. (2023). TMR-Array-Based Pipeline Location Method and Its Realization. Sustainability.

[9] Bian S., Hevesi P., Christensen L., Lukowicz P. (2021). Induced Magnetic Field-Based Indoor Positioning System for Underwater Environments. Sensors.

[10] Raghunath C.R., Prabha V.P., Yeshaswini H.S., Rashmi H., Sushma T.N. (2011). High Performance Interconnection Technology in Avionics (Short Communication). Def. Sci. J..

[11] Chen W.J., Yang Y.Q., Zhang X.D., Zheng Y.J., Zhang B., Yang J., Wang S.M., Sun G.Z., Yuan J.D., Yang D.L. (2022). Research on automatic positioning technology for magnetic field measurement of multipole magnet harmonic coil in heavy ion accelerator. Radiat. Eff. Defects Solids.

[12] Zhang Q., Li Y., Xu H., Li X., Zhang X. (2023). Magnetic Localization Method of Capsule Endoscope Based on Hybrid Model. IEEE Trans. Instrum. Meas..

[13] Lee S., Kim H., Son H. (2022). Moment method based distributed multipoles for modeling magnetic materials in 2D and 3D magnetostatics. Comput. Methods Appl. Mech. Eng..

[14] Ren Y., Hu C., Xiang S., Feng Z. (2015). Magnetic dipole model in the near-field. 2015 IEEE International Conference on Information and Automation.

[15] Ouyang G., Abed-Meraim K., Ouyang Z. (2023). Magnetic-Field-Based Indoor Positioning Using Temporal Convolutional Networks. Sensors.

[16] Sasaki A. (2022). Effectiveness of Artificial Neural Networks for Solving Inverse Problems in Magnetic Field-Based Localization. Sensors.

[17] Fu Q., Zhao D., Shao L., Zhang S. (2024). Magnetic Localization Algorithm of Capsule Robot Based on BP Neural Network. IEEE Trans. Instrum. Meas..

[18] Lin C., Liu H., Li X., Fu Q., Chen P. The Magnetic Positioning System Based on Genetic Algorithm-optimized BP Natural Network. Proceedings of the 2024 IEEE 25th China Conference on System Simulation Technology and Its Application (CCSSTA).

[19] Qiu C., Qian Z., Qi Q., Wang R., Li X., Bai R. (2025). AI-Assisted Passive Magnetic Distance/Position Sensor. Sensors.

[20] Wu F.Y., Foong S., Sun Z. (2015). A Hybrid Field Model for Enhanced Magnetic Localization and Position Control. IEEE/ASME Trans. Mechatron..

[21] Sun D., Wei E., Yang L., Xu S. (2020). Improving Fingerprint Indoor Localization Using Convolutional Neural Networks. IEEE Access.

[22] Sasaki A., Ohta E. (2020). Magnetic-Field-Based Position Sensing Using Machine Learning. IEEE Sens. J..

[23] Wang M., Shi Q., Song S., Meng M.Q.-H. (2020). A novel magnetic tracking approach for intrabody objects. IEEE Sens. J..

[24] Song S., Hu C., Li M., Yang W., Meng M.Q.-H. (2009). Two-magnet-based 6D-localization and orientation for wireless capsule endoscope. IEEE International Conference on Robotics and Biomimetics (ROBIO).

